# Supplementary material for: MetaRibo-Seq measures translation in microbiomes
Source: Nat Commun. 2020 Jun 29;11:3268. doi: 10.1038/s41467-020-17081-z (PMC7324362; doi:10.1038/s41467-020-17081-z)
Supplement: Supplementary file 10 — Supplementary Data 7 [file 41467_2020_17081_MOESM10_ESM.zip › File2/Confidence_High_Taxonomy/290608_out.krona.html]

Javascript must be enabled to view this page.

members
magnitude
magnitudeUnassigned
count
unassigned
taxon
rank

290608\_out

118

superkingdom
118
2

976
118
phylum

200643
class
118

171549
118
order

815
118
family

816
118
genus


SRS014235\_contig\_number\_49533SRS015890\_contig\_number\_3836SRS015890\_contig\_number\_12472SRS016954\_contig\_number\_27537SRS017521\_contig\_number\_31610SRS018133\_contig\_number\_9227SRS018984\_contig\_number\_5777SRS019161\_contig\_number\_23869SRS019178\_contig\_number\_contig-100\_292.42662SRS019445\_contig\_number\_35252SRS019685\_contig\_number\_7720SRS024492\_contig\_number\_3472SRS042284\_contig\_number\_4223SRS044535\_contig\_number\_22308SRS046502\_contig\_number\_780SRS047014\_contig\_number\_4503SRS049402\_contig\_number\_4250SRS056273\_contig\_number\_33195SRS058770\_contig\_number\_22802SRS064973\_contig\_number\_1647SRS075078\_contig\_number\_31303SRS076804\_contig\_number\_contig-100\_1899.122912SRS077127\_contig\_number\_15784SRS077730\_contig\_number\_contig-100\_1551.113012SRS1041118\_contig\_number\_11015SRS104311\_contig\_number\_32831SRS140492\_contig\_number\_2544SRS147377\_contig\_number\_10023SRS893187\_contig\_number\_12314SRS971275\_contig\_number\_31413SRS971276\_contig\_number\_5240
31
species
371601


SRS017745\_contig\_number\_651
species
1
46506

5
species

SRS016132\_contig\_number\_4173SRS063370\_contig\_number\_99SRS077294\_contig\_number\_7694SRS097889\_contig\_number\_18355SRS893252\_contig\_number\_12711
2292951


SRS050299\_contig\_number\_2110
species
1
246787

1739517
species
1

SRS1041141\_contig\_number\_12292

28116
79
species

SRS011084\_contig\_number\_20870SRS011302\_contig\_number\_15105SRS013521\_contig\_number\_6438SRS013687\_contig\_number\_20465SRS014459\_contig\_number\_29141SRS014979\_contig\_number\_contig-100\_2079.181391SRS015065\_contig\_number\_24119SRS015264\_contig\_number\_21409SRS015369\_contig\_number\_1210SRS015431\_contig\_number\_84643SRS015663\_contig\_number\_6083SRS015694\_contig\_number\_23698SRS015960\_contig\_number\_18522SRS016203\_contig\_number\_8523SRS016840\_contig\_number\_contig-100\_469.33848SRS016989\_contig\_number\_5896SRS017103\_contig\_number\_42193SRS017247\_contig\_number\_8592SRS017307\_contig\_number\_6277SRS017821\_contig\_number\_33513SRS019068\_contig\_number\_82676SRS019381\_contig\_number\_4001SRS019968\_contig\_number\_13625SRS021219\_contig\_number\_contig-100\_167.75747SRS021484\_contig\_number\_21986SRS022713\_contig\_number\_2124SRS022713\_contig\_number\_5956SRS023583\_contig\_number\_17338SRS023914\_contig\_number\_28902SRS024075\_contig\_number\_16315SRS024331\_contig\_number\_31829SRS046369\_contig\_number\_15476SRS049959\_contig\_number\_48210SRS049995\_contig\_number\_contig-100\_3347.181522SRS050925\_contig\_number\_6605SRS050941\_contig\_number\_19345SRS052027\_contig\_number\_5942SRS052697\_contig\_number\_35813SRS053398\_contig\_number\_3582SRS056259\_contig\_number\_22576SRS057478\_contig\_number\_2194SRS058268\_contig\_number\_contig-100\_394.395SRS062654\_contig\_number\_27005SRS063813\_contig\_number\_5009SRS064557\_contig\_number\_3001SRS065504\_contig\_number\_38174SRS076756\_contig\_number\_18741SRS076976\_contig\_number\_6661SRS077024\_contig\_number\_contig-100\_1913.81267SRS077086\_contig\_number\_1063SRS077194\_contig\_number\_15899SRS078242\_contig\_number\_contig-100\_1717.179665SRS098061\_contig\_number\_3069SRS098644\_contig\_number\_23176SRS1041092\_contig\_number\_10867SRS1041095\_contig\_number\_5617SRS1041147\_contig\_number\_1868SRS104400\_contig\_number\_48797SRS104975\_contig\_number\_9429SRS105153\_contig\_number\_27192SRS142503\_contig\_number\_57926SRS143372\_contig\_number\_1268SRS144183\_contig\_number\_41370SRS144506\_contig\_number\_10358SRS144537\_contig\_number\_37547SRS144603\_contig\_number\_7697SRS147088\_contig\_number\_contig-100\_140.42400SRS147346\_contig\_number\_35638SRS147425\_contig\_number\_5184SRS148319\_contig\_number\_14496SRS148511\_contig\_number\_2071SRS149075\_contig\_number\_15165SRS149181\_contig\_number\_10567SRS149244\_contig\_number\_6961SRS893170\_contig\_number\_8468SRS893253\_contig\_number\_927SRS893256\_contig\_number\_400SRS893378\_contig\_number\_3497SRS893382\_contig\_number\_1313
